# Identification of a Mitochondrial Target of Thiazolidinedione Insulin Sensitizers (mTOT)—Relationship to Newly Identified Mitochondrial Pyruvate Carrier Proteins

**DOI:** 10.1371/journal.pone.0061551

**Published:** 2013-05-15

**Authors:** Jerry R. Colca, William G. McDonald, Gregory S. Cavey, Serena L. Cole, Danielle D. Holewa, Angela S. Brightwell-Conrad, Cindy L. Wolfe, Jean S. Wheeler, Kristin R. Coulter, Peter M. Kilkuskie, Elena Gracheva, Yulia Korshunova, Michelle Trusgnich, Robert Karr, Sandra E. Wiley, Ajit S. Divakaruni, Anne N. Murphy, Patrick A. Vigueira, Brian N. Finck, Rolf F. Kletzien

**Affiliations:** 1 Metabolic Solutions Development Company, Kalamazoo, Michigan, United States of America; 2 Southwest Michigan Innovation Center, Kalamazoo, Michigan, United States of America; 3 Medros, Inc., St. Louis, Missouri, United States of America; 4 Department of Pharmacology, University of California San Diego, La Jolla, California, United States of America; 5 Department of Medicine, Washington University School of Medicine, St. Louis, Missouri, United States of America; University of Texas Health Science Center at San Antonio, United States of America

## Abstract

Thiazolidinedione (TZD) insulin sensitizers have the potential to effectively treat a number of human diseases, however the currently available agents have dose-limiting side effects that are mediated via activation of the transcription factor PPARγ. We have recently shown PPARγ-independent actions of TZD insulin sensitizers, but the molecular target of these molecules remained to be identified. Here we use a photo-catalyzable drug analog probe and mass spectrometry-based proteomics to identify a previously uncharacterized mitochondrial complex that specifically recognizes TZDs. These studies identify two well-conserved proteins previously known as brain protein 44 (BRP44) and BRP44 Like (BRP44L), which recently have been renamed Mpc2 and Mpc1 to signify their function as a mitochondrial pyruvate carrier complex. Knockdown of Mpc1 or Mpc2 in *Drosophila melanogaster* or pre-incubation with UK5099, an inhibitor of pyruvate transport, blocks the crosslinking of mitochondrial membranes by the TZD probe. Knockdown of these proteins in *Drosophila* also led to increased hemolymph glucose and blocked drug action. In isolated brown adipose tissue (BAT) cells, MSDC-0602, a PPARγ-sparing TZD, altered the incorporation of ^13^C-labeled carbon from glucose into acetyl CoA. These results identify Mpc1 and Mpc2 as components of the mitochondrial target of TZDs (mTOT) and suggest that understanding the modulation of this complex, which appears to regulate pyruvate entry into the mitochondria, may provide a viable target for insulin sensitizing pharmacology.

## Introduction

Diabetes is an undertreated epidemic with an estimated 340 million diabetic persons world-wide and rapidly increasing incidence throughout the developed and developing world [1]. Thiazolidinedione (TZD) insulin sensitizers have proven to be effective therapeutic agents for treating a root cause of diabetes, namely insulin resistance, and also have been shown to preserve the function of the pancreatic β-cells, thus preventing progression of pre-diabetic patients to frank disease [2], [3]. However, in spite of considerable effort, no regulatory approvals of new insulin sensitizing agents have occurred over the past decade [4], [5]. TZD pharmacology has generally been thought to require direct activation of the nuclear transcription factor PPARγ and, since it is now generally accepted that activation of PPARγ drives the side effects of this class of agents (e.g., increased adiposity, volume expansion, bone loss and congestive heart failure), there has been no clear path forward to create new, more useful insulin sensitizers [4]. Recently, Chen *et al.* have shown that TZD analogs that do not bind or activate PPARγ at physiologic concentrations have a very similar insulin sensitizing pharmacology as produced by rosiglitazone and pioglitazone in obese rodent models [6]. Moreover, TZDs also exerted insulin-sensitizing effects in isolated hepatocytes that were completely independent of PPARγ as shown by using hepatocytes from liver-specific PPARγ knockout mice [6]. PPARγ-sparing TZDs are now in Phase II clinical trials and a prototype compound has been shown to lower glucose to the same extent as pioglitazone in diabetic patients with reduced side effects associated with activation of PPARγ [7]. These findings suggest that other mechanisms through which insulin sensitizers signal are involved in their anti-diabetic pharmacology. The discovery of novel insulin sensitizing agents would be facilitated if an alternate TZD target could be identified and its biological function established.

Numerous investigators have suggested that TZDs may have important effects on mitochondrial function and/or biogenesis (reviewed in [8]). Indeed, we showed several years ago that a mitochondrial binding site for TZDs existed and we have hypothesized that this may be important for the efficacy of this class of drug [9]. We have designed a photo-catalyzable affinity probe based on the structure of pioglitazone. This technique provides a molecular tool that once selectively bound to its site of action, can be covalently attached (crosslinked) to the site. Demonstration of specificity is by selective competition prior to the photo-activation of the probe [10]. We have employed this technique along with mass spectrometry-based proteomics to identify BRP44, a protein in the inner mitochondrial membrane that is selectively crosslinked by this probe. BRP44 is an evolutionarily-conserved protein and crosslinking studies of the BRP44 ortholog in *Drosophila* revealed the presence of a second related protein, BRP44 Like, which is also required for the crosslinking by TZDs. Immunoprecipitation studies suggest that both of these proteins form a multi-subunit complex that could provide a new target for designing insulin sensitizers. Importantly, two recent publications have independently demonstrated that these same proteins, which have been renamed, Mpc1 and Mpc2 (BRP44 Like and BRP44, respectively), are key components of the mitochondrial pyruvate carrier complex [11], [12], placing these proteins and the mitochondrial action of the insulin sensitizers at a critical nodal point of metabolism.

## Materials and Methods

### Materials

The photoaffinity crosslinker and all TZDs were synthesized at Kalexsyn (Kalamazoo, MI). The photoaffinity crosslinker was iodinated with carrier-free ^125^I (Perkin-Elmer) using Iodogen (Pierce), purified on a C_18_ column and stored in the dark at −20°C as previously described [9].

### Membrane isolation and drug analog photoaffinity crosslinking

All animal handling took place at Western Michigan University. Tissue collection was carried out following carbon dioxide asphyxiation and exsanguination out in strict accordance with the recommendations in the Guide for the Care and Use of Laboratory Animals of the National Research Council. The protocol was approved by the Institutional Animal Care and Use Committee of Western Michigan University (Animal Use Protocol Number: 10-01-02). Crude mitochondrial membranes were prepared by differential centrifugation of homogenized mouse tissues followed by photoaffinity crosslinking as previously described [9]. Mouse liver mitochondrial fractions were prepared as previously described [13] by treating with 0.15 mg digitonin/mg of crude mitochondria in fractionation buffer (FB: 250 mM sucrose, 10 mM Tris, pH 8, and Roche complete protease inhibitor cocktail). Separation of the soluble outer membrane and inner membrane space from the mitoplast pellet was accomplished by centrifugation at 9500× g. Resulting mitoplasts were treated with 0.5 mg Lubrol/mg protein in FB on ice for 15 minutes. The lubrol treated samples were placed on top of a 3 step gradient of 51%, 37% and 23% sucrose in 20 mM Na_2_PO_4_, pH 7.4 containing protease inhibitors and centrifuged at 100,000× g in a Beckman SW 32Ti rotor. Following centrifugation the matrix fraction was removed from the top gradient and the inner membrane fractions were collected from the 51 and 37% interfaces. Crosslinked material was separated by either SDS-PAGE and then evaluated by Western blotting or initially by Blue Native gel separation followed by a second dimension separation on SDS-PAGE. The gels were dried and exposed to X-ray film to identify the ^125^I-labled proteins. In some cases the crosslinking was carried out in tissues from mitoNEET null mice [14].

### Identification of proteins by proteomics

Crosslinked liver mitochondrial samples (20,000× g) were solubilized in 1% n-Dodecyl β-D-Maltopyranoside (DMM) and ATP synthase related peptides were removed by immunoprecipitation before separation of the enriched sample by Blue Native electrophoresis. The first dimension Blue Native gels were then further resolved by SDS-PAGE to identify the specifically crosslinked proteins, which were located by the satellite silver stained patterns. Specifically crosslinked bands were cut from the gel, reduced, alkylated, and trypsin digested in situ and subjected to nanoLC-MS/MS [9]. Samples were concentrated and desalted using on-line trapping then separated on a 75 µm×10 cm BEH column (Waters) using a nanoAcquity UPLC system coupled to a Q-Tof Premier mass spectrometer. Data for protein identification was acquired in MS∧E mode and searched against a NCBI and SwissProt databases using Waters Proteinlynx Global Server software v2.4.

### Generation of antibodies and Western blotting

Rabbit antibodies were prepared against synthetic peptides AYHYHQSQEKLKQEQQQPAV and SSTAVQPPPPVPPPPPSAVP for GC9399 and TNATAQSIQGLRFLHYNYGS and SIRRAMSTTASKEWRDYFMS for CG14290 *Drosophila* sequences, respectively. Rabbit antibodies were prepared against KLRPLYNHPAGPRTVFFWA of the mammalian sequence of BRP44. BRP44L antibodies were obtained from Sigma. UCP1 antibodies were obtained from Abcam. Antibodies to the His-Tag were obtained from Cell Signaling.

### Expression of C-terminal extended BRP44

HEK293 cells were plated twenty-four hours prior to transfection in 6-well plates at 6×10^5^ cells/well in 2 mls/well antibiotic free DMEM (Gibco 11965) supplemented with 10% Fetal Bovine Serum (Gibco 16000), 1∶100 MEM Non-Essential Amino Acids (Gibco 11140), 1∶100 Sodium Pyruvate (Gibco 11360) and 1∶100 GlutaMAX (Gibco 35050) or in 100 mm dishes at 3.5×10^6^ cells each in 10 mls. Plasmid DNA for transfection was purified using the Clontech Nucleobond PC 500 kit (740574.25). Cells were transfected using Lipofectamine 2000 Transfection Reagent (Invitrogen 11668) at a 1∶1 ratio of DNA (µg) to Lipofectamine (µl) following Invitrogen's suggested transfection protocol. Complexes were formed in Opti-MEM I Reduced Serum Medium (Gibco 11058-021) by diluting the appropriate amounts of DNA and Lipofectamine separately into Opti-MEM I, incubating at room temperature for 5 minutes, combining DNA dilutions with Opti-MEM I dilutions and incubating another 20 minutes at room temperature before adding transfection complexes dropwise to wells and plates. Plating medium was not removed or exchanged prior to transfection. The 6-well dishes were transfected using 5 µg of DNA and 5 µl Lipofectamine in 500 µl Opti-MEM I per well. The 100 mm plates were transfected using 30 µg DNA and 30 µl Lipofectamine in 3 mls Optim-MEM I per plate. The medium was not replaced after transfection.

### *Drosophila* studies

*D. melanogaster w^1118^* strain was used for initial crosslinking experiments. 3rd instar larvae and 3–4 days old flies were collected. Larvae were washed in PBST. Tissues were processed, and crosslinked as described above. Ubiquitous GAL4 driver y^1^ w*; P{Act5C-GAL4}17bFO1/TM6B, Tb^1^ (stock 3954) was obtained from Bloomington Drosophila Stock Center. RNAi knock-down lines targeting CG9399: *w^1118^; P{GD4636}v13788* (stock v13788), *w^1118^; P{GD4636}v13789* (stock v13789); targeting CG9396 *P{KK112662}VIE-260B* (stock v104068); targeting CG32832 *w^1118^; P{GD15967}v47810* (stock v44810), *w^1118^; P{GD15967}v47811/TM3* (stock v47811), *P{KK113045}VIE-260B* (stock v102996); targeting CG14290 *w^1118^; P{GD3944}v15858* (stock v15858), *P{KK102734}VIE-260B* (stock v103829); targeting CG16790 *w^1118^; P{GD8781}v19160* (stock v19160), *P{KK107622}VIE-260B* (stock v104333); and parental strains v60000, and v60100 were obtained from Vienna Drosophila Resource Center. Act-GAL4 driver was crossed to RNAi construct carrying lines; progeny not carrying a balancer chromosome was used for assays. Progeny (non-balanced) from the cross between Act-GAL4 and corresponding parental line (60000 for GD lines and 60100 for KK lines) served as the control.

Flies were raised on semi-defined media (BDRC recipe). Normal (Low) Sugar media contained 0.15 M sucrose. High Sugar media had 0.75 M sucrose. Fly eggs were laid on the media surface and hatching larvae were feeding the media of choice. Eclosed adult flies (females) were transferred to vials with fresh media within 24 h and aged for 7 days. Hemolymph glucose measurements were performed as follows: adult female flies were collected and punctured in the thorax with 25G syringe needle and hemolymph was collected by centrifugation at 4,500× g for 1 min. Samples were processed using Infinity glucose (Ox) reagent (Thermo Scientific) according to manufacturer instructions. Full genome expression arrays were obtained from Agilent. Slides hybridization, reading, and basic statistical analysis were done at MOGene (St Louis). Three biological replicas were obtained for each experimental condition. Insulin-stimulated increase in pAKT was performed as previously described [15]. Antibodies to AKT and phosphor AKT (Ser505 homologous to human Ser473) were from Cell Signaling.

### Brown adipose progenitor cell differentiation assay

Brown adipose progenitor cells were isolated from the interscapular pad of CD1 mice (Charles River) and cultured in 35 mm dishes according to the method of Petrovic *et al.* [16]. When the progenitor cells reached 90% confluence, differentiation was initiated by treating the cells for 6 days with increasing concentrations of TZDs or non-TZDS in DMEM containing 25 nM insulin and 10% FBS. UCP1 Western analysis was conducted on cell lysates followed by densitometry on the immunoreactive bands using Image J software.

### ^13^C Glucose Flux in primary brown adipose cells

Brown adipose progenitor cells were isolated as described above. When the progenitor cells had expanded to 90% confluence, differentiation was initiated by treating the cells for 48 hours with 1 µM MSDC-0160 in DMEM containing 25 nM insulin and 10% FBS. The cells were then switched to DMEM containing 25 nM insulin and 10% FBS with no drug for 96 hours. Flux was conducted on the differentiated cells by pretreating for 2 hours in DMEM containing 5 mM glucose, 10% FBS, 25 nM insulin and increasing concentrations of drug. The 15 minute flux experiments were initiated by removing the DMEM, rinsing 1X with serum free DMEM and adding KRHB (Krebs Ringer-HEPES Buffer) with 5 mM ^13^C-glucose (U-^13^C; Cambridge Isotope Laboratories, Inc.), 25 nM insulin and drug. The flux reaction was quenched by rinsing once with ice cold PBS, once with ice cold water, and extracting with dry-ice chilled methanol∶chloroform as previously described [17].

### LC-MS analysis of Acetyl CoA

Metabolomic analysis was performed using a Waters quadrupole time-of-flight (Qtof Premier) mass spectrometer mode coupled to a Waters nanoAcquity UPLC. TCA cycle intermediates were separated using ion pairing reverse-phase chromatography with a Waters BEH C-18 column (1.7 µm particle, 200 µm ID ×10 cm length) and detected in the negative ion mode. The mobile phase A was 10 mM tributylamine, 15 mM acetic acid in 97∶3 water∶methanol and mobile phase B was methanol. The flow rate was 2 µL/min and the gradient was 0 min 0.5% B; 2.5 min 0.5% B; 19 min 95% B; 23 min 95% B; 26 min 0.5% B. Waters Masslynx software was used to collect centroid, lockmass corrected data. Entry of ^13^C was measured by comparing the ratio of resolved isotopes of acetyl-CoA. Data were analyzed using Waters Quanlynx software to determine the relative abundance of selected ions.

Numerical data are presented as mean and standard error and statistical comparison have been made by an unpaired, two-tailed t-test to derive p values shown.

## Results

### Identification of a mitochondrial protein crosslinked selectively by the TZD photoprobe

**Figure 1:**
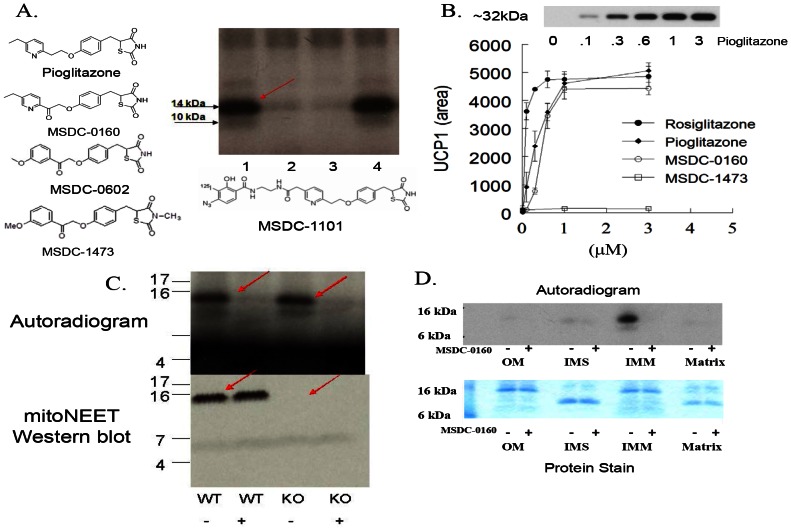
Selective crosslinking with photo affinity probe. (**A**) Mitochondrial membranes (20 µg) from rat liver were incubated with the iodinated (^125^I) photo-probe (MSDC-1101) in the absence of any competing compound (lane 1); in the presence of 25 µM MSDC-0160 (lane 2); in the presence of 25 µM MSDC-0602 (lane 3); or in the presence of 25 µM MSDC-1473 (lane 4). Following exposure to UV light, samples were separated on one dimensional SDS-PAGE and the dried gel was exposed to X-ray film. (**B**) Active TZDs, but not MSDC-1473, produce a dose-dependent increase in UCP1 as detected on Western blots from mouse BAT progenitor cells. The abscissa shows drug concentration (µM). The inset on the top of this figure shows a representative Western blot of the increase in UCP1 protein in cells treated with an active TZD (pioglitazone). The data below the blot show the dose dependent increases observed in a representative experiment from a scan of the Western blots (arbitrary units, Mean and SE; N = 3). (**C**) Liver mitochondrial fractions from wild type or mitoNEET null mice [11] were crosslinked as in A without (−) or with (+) 25 µM MSDC-0160. The top figure is the resulting autoradiogram and the bottom is the Western blot for mitoNEET. (**D**) Mouse liver mitochondria were fractionated to generate fractions for outer membrane (OM), inter membrane space (IMS), inner membrane (IMM), and matrix. Total protein (10 µg) from each fraction was crosslinked as in A. The autoradiogram is shown at the top and a stain for total protein for the same 4 mitochondrial subfractions is shown below.

Incubation of the photoprobe, MSDC-1101, with mouse liver mitochondrial membranes followed by photoactivation revealed a ∼14 kDa radiolabeled band on the film (Figure 1A, see arrow, lane 1). The addition of active TZDs such as MSDC-0160 and MSDC-0602 before photoactivation inhibited crosslinking (lanes 2 and 3). In contrast, the addition of a TZD analog in which a methyl group has been incorporated on the thiazolidine ring nitrogen (MSDC-1473), resulted in no inhibition of crosslinking (Figure 1A, lane 4). Unlike the other analogs, MSDC-1473 is inactive in cellular assays, such as the activation of UCP1 expression in brown adipocyte precursor cells (Figure 1B). The specificity of these competitive interactions for crosslinking versus the activity in cells suggests that this mitochondrial protein could be an important drug target.

Earlier efforts to identify the crosslinked protein suggested that it was mitoNEET, a protein in the outer mitochondrial membrane [8], [14]. However, we now show that probe crosslinking of mitochondrial membranes was not reduced in the complete absence of mitoNEET protein in liver (or muscle, not shown) from mitoNEET null mice (Figure 1C). Moreover, the crosslinked protein was localized to the inner mitochondrial membrane (Figure 1D) while mitoNEET is an outer mitochondrial membrane protein [14].

**Figure 2:**
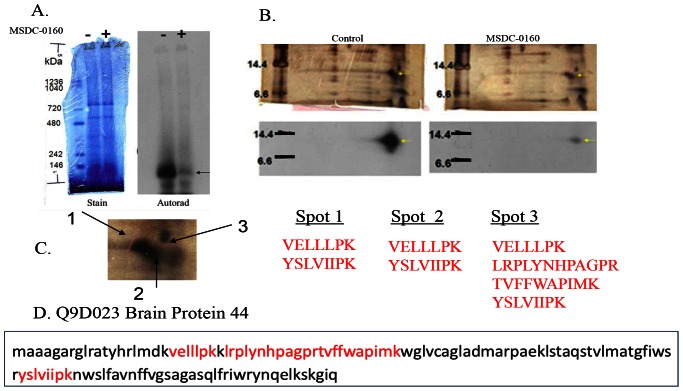
Crosslinked protein is BRP44. (**A**) Blue Native electrophoresis of crosslinked proteins showing the stain pattern (left) and the corresponding autoradiogram image for the specifically crosslinked protein (without, “−”on the left and with MSDC-0160 competition, “+”, on the right). The <150 kDa band is marked by the arrow. (**B**) Second dimension SDS separation of Blue Native gel without (left) and with (right) MSDC-0160 competition. Upper figures are silver stained images of the gels and lower figures are corresponding autoradiograms. Areas of interest are marked by the yellow arrows. (**C**) Expanded view of silver-stained images showing the three areas submitted for MS/MS analysis followed by the respective trypsin peptide sequences. These peptides (shown also in red, bottom frame **D**) identify BRP44 (Q9D023).

The radiolabeled protein was successfully purified by a three step approach involving solubilization and immunoprecipitation to remove ATP synthase and related binding proteins. This was followed by Blue Native gel electrophoresis in which the crosslinked protein ran as a ∼150 kDa complex (Figure 2A) from which a second dimension SDS-PAGE produced a single, selectively radiolabeled protein (final gel products are shown in Figure 2B). The silver stain of the gel from a sample prepared in the absence of competing analog is shown on the left and one from a sample with the competing analog is shown on the right. Beneath each silver stained gel is the image from the X-ray film from the sample. The position of the specifically crosslinked protein is shown by the yellow arrow on the X-ray image and the silver stained gel. Figure 2C shows an enlarged region of interest on the silver stained gels. The specific radiolabeled regions overlay two silver-stained spots (spots 1 and 2) and a third area of the gel (spot 3) that did not stain with silver is marked by the arrows. These areas were cut from the gels and subjected to trypsin digestion and MS/MS analysis.

**Figure 3:**
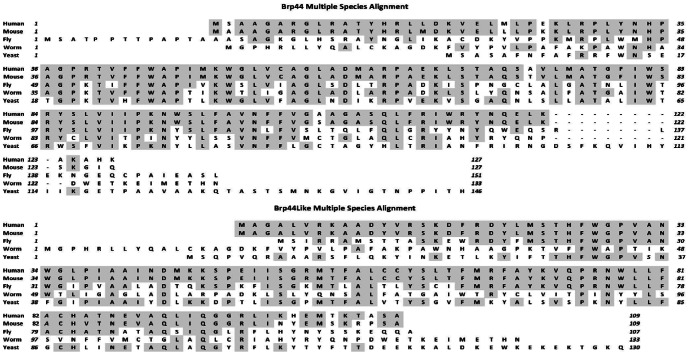
BRP44 family. Upper panel (A) is the multiple species sequence alignment of BRP44 and lower panel (B) shows the multiple species alignments for the family member protein BRP44L. Shaded amino acids show identical amino acids as compared to the human sequences.

All three gel slices analyzed by mass spectrometry identified 2–4 tryptic peptides from BRP44 (Q9D023) (Figure 2D). Several spots from the gel also contained some tryptic peptides of microsomal glutathione S transferase, however this protein could be completely removed from the solubilized crosslinked sample on a glutathione column without trapping the labeled protein (not shown). Thus, the crosslinked protein seemed to be the sequence identified in the data base as BRP44 (Q9D023-Figure 2D) a phylogenetically well-conserved protein (Figure 3A), which is a member of a small protein family that includes BRP44L, also phylogentically conserved (Figure 3B).

**Figure 4:**
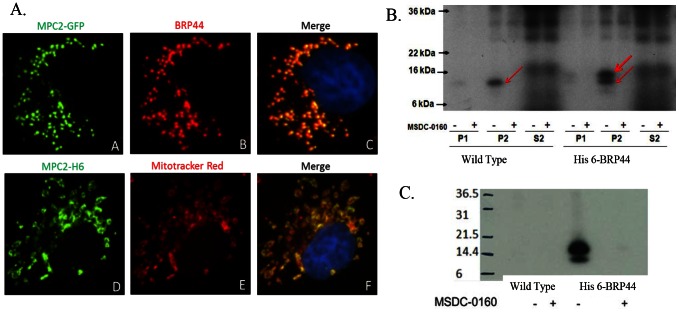
Expressed BRP44 goes to mitochondrion and is crosslinked selectively by the TZD probe. (**A**) Upper frame: C-terminally-tagged GFP BRP44 (green) in Cos7 cells is co-localized with endogenous BRP44 (red). Lower frame: C-terminally tagged 6×His BRP44 (green) is co-localized with Mitotracker Red confirming the mitochondrial localization. (**B**) A sequence encoding BRP44 C-terminally tagged with six histidine residues was transiently expressed in HEK293 cells. Subcellular fractions were prepared (P1, crude nuclear fraction; P2, crude mitochondrial fraction; S2, post mitochondrial fraction) and incubated with the radiolabeled photo-probe in the presence or absence of 25 µM MSDC-0160 as indicated. Samples were subjected to one dimensional SDS-PAGE and the dried gel was exposed to X-ray film. The red arrows point to the image of the selectively crosslinked native and C-terminal extension proteins. (**C**) Crosslinked BRP44 6×His is selectively immunoprecipitated by antibodies to 6×His. Membranes from HEK293 cells stably transfected with BRP44 6×His (right lanes, 6 His) or non transfected cells (left lanes, control) were crosslinked with or without competition with MSDC-0160 as in B. The membranes, all of which contained the specifically crosslinked band as shown in B, were then solubilized and immunoprecipitation was carried out with anti-His agarose beads. The resulting pellets were solubilized and submitted to SDS-PAGE. The corresponding autoradiogram is shown demonstrating that the radioactive band is only precipitated by anti-His tag in the tissue from the cells expressing His-6 BRP44.

BRP44 was cloned and expressed in Cos7 cells with a C-terminal GFP tag or a 6×His tag. The native protein and the GFP fusion protein were co-localized and an overlay with Mitotracker Red revealed that the BRP44 protein is exclusively localized in the mitochondria of these cells (Figure 4A). Subcellular fractions from cells that expressed the 6×His tag BRP44 were subjected to photoaffinity crosslinking resulting in both the native protein and the larger his-tagged species being selectively crosslinked (Figure 4B). In a separate set of experiments, solubilized drug-affinity crosslinked (with MSDC-1101) mitochondria from HEK293 cells stably expressing BRP44 with a C-terminal 6×His-tag were immunoprecipitated with an antibody to 6×His. The resulting autoradiogram (Figure 4C) demonstrated that the crossked protein was precipitated while no labeled proteins were precipitated from the wild type cells. This precipitated both the radiolabled 6×His-tagged BRP44 as well as other proteins in the complex, including the native BRP44 and family member BRP44-Like (demonstrated by Western blots and mass spectrometry, not shown). These data provide strong evidence that BRP44 is the mitochondrial protein that is crosslinked by the TZD photoprobe.

**Figure 5:**
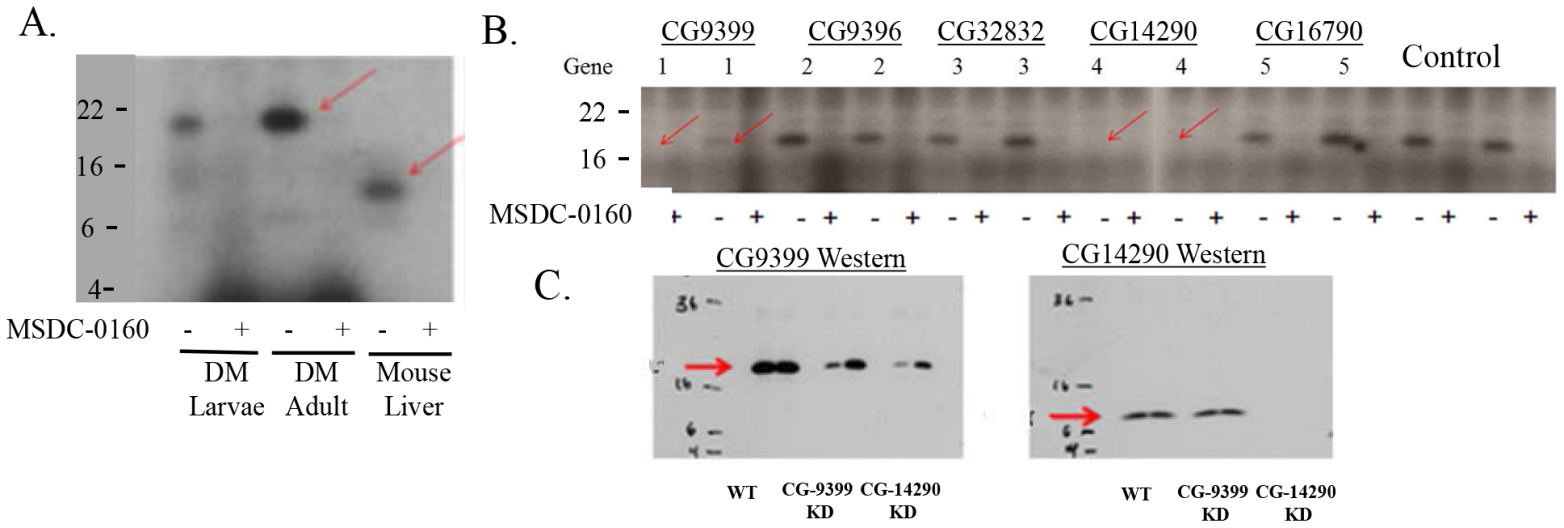
Crosslinking of the *Drosophila* ortholog in wild type and knockdown lines. (**A**) Wild-type larvae and adult flies were homogenized and mitochondrial pellets were crosslinked with and without the addition of MSDC-0160 as in figure 1. The autoradiograms of the resulting image of the specifically crosslinked proteins are shown for the flies on the left side compared to mouse liver in the lane on the right. (**B**) Fly stocks carrying RNAi constructs were crossed to Act-GAL4 driver and progeny tissues were processed and crosslinked as described in the text. Lanes marked “1” were from knockdowns of CG9399, lanes marked “4” were from knockdowns of CG14290, the other lanes were either unrelated genes or control strains. Two separate knockdown lines are represented. The complete list of the strains is shown in the text. Red arrows indicate reduced crosslinking of the specific CG9399 protein (BRP44 ortholog). (**C**) Membrane samples from CG9399 and CG14290 KD as in B were subjected to Western blot analysis for either the CG9399 (left) or CG14290 protein (right).

Given the phylogenic conservation of BRP44 (Figure 3A) and the genetic tractability of a model organism, we evaluated whether the *Drosophila* ortholog would show an interaction with the TZD probe. As shown in Figure 5A, the TZD photoprobe specifically crosslinked a protein in crude mitochondrial pellets from both larvae and adult flies. This is the expected size for the Drosophila BRP44 ortholog, CG9399, which has an N-terminal extension relative to the murine and human orthologs (Figure 3A). Database searches identified four additional *Drosophila* proteins with significant sequence homology and fly lines with RNAi-mediated knockdown of these genes were obtained. As predicted from analyses of these sequences, the knockdown of CG9399 (gene 1, Figure 5B) reduced the level of crosslinking in two fly lines. Knockdown of CG9396, CG32382, or CG16790 had no effect on the crosslinking, however, the knockdown of a CG14290, which was predicted to code for the BRP44 Like ortholog (Figure 5B, lanes labeled 4) also reduced the crosslinking. The BRP44 Like protein is also well-conserved (Figure 3B). This result suggests that these two proteins might function together to create the binding site recognized by the TZDs. Antibodies were made to both proteins and utilized in Western blots to confirm the knock down of the proteins (Figure 5C). These data demonstrate that the knockdown of the expression of CG14290 protein (the BRP44 Like ortholog) also reduced the protein levels of CG9399 (BRP44 ortholog), which could explain the reduced crosslinking of the CG9399 protein with the knockdown of the expression of either gene.

### Mechanistic Evaluation of Pyruvate Metabolism

**Figure 6:**
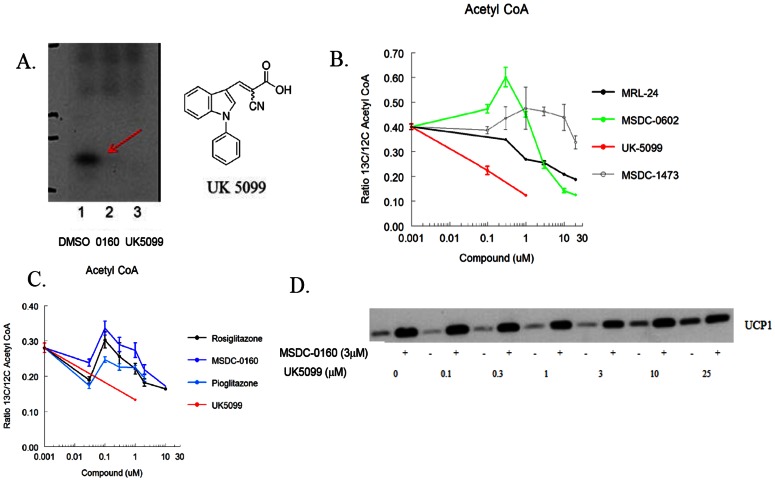
BPR44 and BRP44L are involved in pyruvate transport. (**A**) UK5099 structure and effect of adding either 25 µM MSDC-0160 (lane 2) or UK5099 (lane 3) on crosslinking of BRP44 (Mpc2). Lane 1 is the DMSO control. (**B**) Incubation of mouse BAT cells with UK5099 effectively limits carbon flow from U-^13^C glucose into acetyl CoA (red line) while MSDC-0602 has a biphasic dose response. The non TZD insulin sensitizer MRL-24 also modulated flow of carbon into acetyl CoA under these conditions. Data are the ratio of Acetyl CoA (^13^C/^12^C) derived from U-^13^C glucose following a 2 hour pre-incubation and a 15 minute flux at 37C (n = 3; mean and SE; representative of 3 experiments) as described in the Methods. (**C**) A separate experiment similar to that shown in B with other TZDs. (**D**) BAT progenitor cells were incubated with the indicated concentrations of UK5099 with (+) or without (−) 3 µM MSDC-0160 for 5 days. Cells were lysed and Western blots were conducted for UCP1 as in Figure 1B.

Bricker *et al.* [11] provided evidence that Mpc1 (BRP44L) may be the site of interaction of the potent pyruvate transport inhibitor α-cyannocinnamate analog, UK-5099. This conclusion was based on a yeast mutant of Mpc1 that is no longer responsive to inhibition by UK-5099. Adding UK-5099 to the assay, as was seen with active TZDs, prevents the crosslinking of BRP44 (Mpc2) by the TZD probe (Figure 6A), confirming that these two classes of compounds bind in close proximity and suggesting that insulin sensitizing TZDs might modulate pyruvate utilization.

Having shown previously that TZDs have demonstrable effects on isolated BAT cells [16], [18], and now suspecting that this may involve an effect on pyruvate metabolism, we asked whether these compounds could modify the entry of carbon into acetyl CoA in these cells. Carbon flow into acetyl CoA was monitored by the incorporation of ^13^C-glucose into acetyl CoA. As expected, the addition of UK-5099, the potent inhibitor of pyruvate into the mitochondrion, potently blocked the influx of heavy carbon into acetyl CoA (Figure 6B). Treatment with MSDC-0602 resulted in a biphasic change in the incorporation of the heavy label into acetyl CoA. Whereas higher concentrations of MSDC-0602 inhibited incorporation, lower concentrations actually increased ^13^C incorporation into acetyl CoA. Pioglitazone, rosiglitazone, and MSDC-0160 have similar effects as observed with MSDC-0602, while MSDC-1473 was ineffective (Figure 6B and C)). Interestingly, the non-TZD insulin sensitizer MRL-24, a compound which also binds to PPARγ without directly activating it [19], inhibited carbon flow into acetyl CoA under these conditions (Figure 6B). We have also found that MRL-24 also increases UCP1 in BAT cells and competes for crosslinking of Mpc2 (data not shown).

To determine whether simply reducing pyruvate flux would mimic TZD action to increase UCP1 expression in brown fat progenitor cells as shown in Figure 1B, we evaluated the effects of UK-5099 under these conditions. The addition of increasing concentrations of UK-5099 to BAT progenitor cells also resulted in an increase in UCP1 content, however, in spite of the fact that it was more potent at inhibiting pyruvate incorporation, much higher concentrations were required than for the TZD to increase expression of UCP1 (Figure 6D), suggesting that a simple reduction in pyruvate transport is not the mechanism that regulates the expression of UCP1 under these conditions.

### Effect of knockdown of Mpc proteins on pharmacology in flies

**Figure 7:**
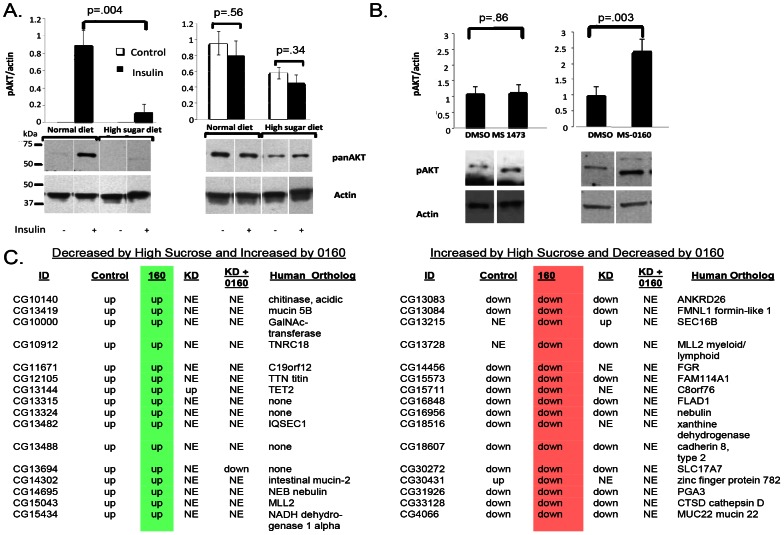
Insulin sensitizer effects in wild type and knockdown flies. (**A**) Acute insulin action was measured in inverted larvae in the absence (−) or presence (+) of 2 µM recombinant insulin. The larvae were either grown under control conditions (left) or in the presence of high sucrose (right). Representative Western blots are shown below and the calculated areas are shown above for pAKT (left side) or total AKT (right side) normalized to actin in the absence (open bars) or presence of insulin (upper bars). An unpaired, two-tailed t-test was used to derive p-values. Error bars are ± SE (N = 3). (**B**) Effect of incubation with DMSO, MSDC-1473 or MSDC-0160 on the amount of insulin-stimulated phosphorylation of AKT under the high sucrose conditions (detected and analyzed as in A; mean and SE, N = 3). (**C**) Wild-type female flies or flies with knockdown of CG14290 (as in Figure 5) were raised on a 0.75M sucrose matrix and aged for 7 days with or without MSDC-0160 added to the matrix. RNA from flies grown under these conditions was extracted and full genome expression analysis was performed using the Agilent system. Conditions in the presence of the high sugar included control flies in addition to knock down of CG14290 (line 15858). MSDC-0160 was provided to either control or line 15859 knockdown flies. The data shown reflect changes that were more than 1.5 fold different and t-test value <9E-03 relative to the high sucrose DMSO control in terms of increase (green) or decrease (red). The list of genes, along with the mammalian ortholog names, are shown on the left in the case of where the expression was decreased in the presence of high sucrose and thus increased with the treatment of MSDC-0160 and on the right for the transcripts that were increased in the presence of high sucrose and thus reduced with the treatment with MSDC-0160. All MSDC-0160-induced changes were lost in the knockdown strain.

Growing Drosophila on a high sucrose medium produces a model of insulin resistance which can be directly demonstrated on insulin signaling in larvae [15]. As shown in Figure 7A, larvae grown on high sucrose matrix demonstrated insulin resistance in terms of the inability of insulin to acutely increase the phosphorylation of AKT. Under these conditions, treatment of the larvae with MSDC-0160 increased insulin action in this respect, while the inactive analog MSDC-1473 was ineffective (Figure 7B). We evaluated the effects of knockdown of the mTOT proteins on the response to high sucrose feeding and insulin sensitizer treatment in this model by evaluating the expression of the genome under these conditions. Expression of 58 genes was increased by the drug treatment and expression of 26 genes was reduced by drug treatment. Importantly, all of these effects were lost upon the knockdown of the Mpc1 ortholog, CG14290, which reduces the expression of both proteins (Figure 5C), suggesting that this complex is involved in the drug action. Figure 7C shows an example of a subset of these genes expressed relative to the differences from the high sucrose control.

## Discussion

In this report, we show that the insulin sensitizing TZDs have a recognition site in the inner mitochondrial membrane that is comprised of a recently identified protein complex involved in mitochondrial pyruvate import. We have called this complex mTOT (**m**itochondrial **t**arget **o**f **t**hiazolidinones). The key members of mTOT, Mpc1 (BRP44 Like) and Mpc2 (BRP44), are highly conserved proteins involved in facilitated pyruvate transport into the mitochondrial matrix [11], [12]. This complex could provide a link between mitochondrial pyruvate metabolism and the efficacy of this class of drugs. Indeed, active insulin sensitizers including pioglitazone and rosiglitazone can directly inhibit pyruvate oxidation and this has also been shown in a variety of intact and permeabilized cells (Divakaruni, unpublished). However the data from figure 6 do not support the hypothesis that the pharmacology is tied directly to limiting pyruvate metabolism *per se*. In fact, it appears that in the intact cells the effects of the compounds on pyruvate flux are complicated and include either an increase or decrease depending on the concentrations of the compounds. Moreover, UK-5099, which is a potent inhibitor of pyruvate flux, did not increase the expression of UCP1 at concentrations at which this inhibition was observed. It will be important to understand more about the other proteins associated with the newly identified mitochondrial complex and to further define the exact effects on insulin sensitizers of both TZD and non TZD classes on the functions of these proteins. The delineation of the mechanisms by which insulin sensitizers modulate metabolism through this interaction may provide insight into the pleiotropic pharmacology of these compounds. These studies may define how post-transcriptional regulation is orchestrated in response to nutritional status and thus provide insight into the potential to treat diseases associated with dysfunctional metabolism. Dysfunctional metabolism is thought to play a role in cancer, neurodegeneration, chronic inflammation, as well as diabetes. Definition of these molecular interactions may also inform design of the next generation of therapeutic agents. This newly identified drug target places insulin sensitizers at the crossroads of carbohydrate, lipid, and amino acid metabolism. Interestingly, alterations in various features of cellular metabolism including fatty acid and amino acid metabolism, are known to be predictors of insulin resistance and to respond to anti-diabetic treatment [20].

Up to the present time, most insulin sensitizer discovery programs have focused on the ability of TZDs and other molecules to bind to and activate the transcription factor PPARγ. This transcription factor is a potent regulator of adipocyte differentiation and many of the original TZD analogs were able to activate this transcription factor to various degrees [21]. Although many active molecules were found by modeling interactions with this target, none of these efforts have yielded a drug that could meet regulatory approval, primarily as a result of unacceptable side effects [4]. Recently, rosiglitazone, the most potent PPARγ activator of the original compounds approved, has essentially been removed from clinical use because of potential cardiovascular side effects [5]. Interestingly, the much weaker PPARγ agonist, pioglitazone remains of clinical use [3]. We had previously suggested that activation of PPARγ is not needed for insulin-sensitizing pharmacology [4], but a plausible alternative mechanistic target has not been provided until this report.

Chen *et al.* [6] demonstrated that novel PPARγ-sparing molecules have similar pharmacology as the first generation insulin sensitizers, rosiglitazone and pioglitazone. In addition, we found that all of the compounds, including rosiglitazone, had clear direct effects on hepatocytes that are completely independent of PPARγ. The studies presented here in *Drosophilia* suggest that the insulin sensitizing pharmacology may require the action of this newly identified mitochondrial complex. Thus, the ability of drug treatment to return the gene expression profile in the presence of high sucrose to the low sucrose control levels was lost upon knockdown of the Mpc proteins (Figure 7C). However, at this time, we do not know the extent to which insulin sensitizing effects are mediated through the mTOT complex in mammals. Firm conclusions await the characterization of genetically modified mice. The critical importance of Mpc1 and Mpc2 expression (both total amount and stoichiometry of the two proteins) to the cell/organism is readily seen in that over-expression in mammalian cells results in cell death (unpublished observations and [22]) while ablation of Mpc2 in mice is embryonic lethal (E11, E12) at a time when mitochondrial function becomes critical for continued development. Thus, studies involving tissue specific, conditional ablation or over-expression will be required to fully characterize the role of these proteins in the regulation of cellular metabolism.

The mitoNEET protein was the first mitochondrial membrane protein identified as a potential mitochondrial insulin sensitizer target based on early studies using the pioglitazone-based photoaffinity probe [9]. This protein has proven of interest to many interested in mitochondrial metabolism [14], [23], [24] and work with the mitoNEET crystal structure has even suggested a potential pioglitazone binding site [24]. However, we have concluded that mitoNEET is not the primary mitochondrial TZD target because we were able to demonstrate photoaffinity crosslinking in liver mitochondrial membranes from mice that were null for expression of mitoNEET (Figure 1). Interestingly, recent work demonstrates that as a component of the outer mitochondrial membrane, mitoNEET can directly bind NADPH [25] and that it plays an important role in the regulation of mitochondrial function and regulation of adiponectin production [26]. An exploration of a possible association of mitoNEET with the mTOT complex is warranted.

In conclusion, this paper identifies mTOT, a mitochondrial target of thiazolidinediones, as a mitochondrial membrane complex involved with pyruvate transport. The cell experiments with ^13^C incorporation into acetyl CoA suggest that the TZDs modulate pyruvate entry into the TCA cycle, however it is not clear from our studies that direct changes in pyruvate flux can itself explain the pharmacology. Thus, the effects of the insulin sensitizers are not mimicked by concentrations of UK-5099 that inhibit pyruvate flux. It is possible that drug interaction may provide a more general signal controlling cell function, especially insulin sensitivity, perhaps by alteration of redox signaling mechanisms. However, Satapati *et al.* have shown that over activation of the TCA cycle is part of the pathology associated with over nutrition and hepatic insulin resistance [27] and this may also contribute to non-alcoholic fatty liver disease [28]. Insulin sensitizers may restore some functions by selectively returning the balance of carbon flow under these conditions. It is unknown at this time whether there are additional functions of Mpc1 (BRP44 Like) and Mpc2 (BRP44) or whether other members of mTOT, the protein complex that contains them, could play important roles in tissue-specific regulation of metabolism and cell function. For example, this complex may regulate mitochondrial fatty acid synthesis or reactive oxygen signals in lower organisms [12], but this has not been examined in mammals. In any event, it is clear these proteins sit at the central control point for regulation of metabolism [29]. The current studies provide background and initial tools for detailed study of this protein complex in metabolic disease and also suggest a new approach for the discovery and development of potentially more useful and novel insulin sensitizers. While this manuscript was in review, studies on direct effects of TZDs on pyruvate metabolism were published [30].

## References

[1] Wild S, Roglic G, Green A, Sicree R, King H (2004) Global prevalence of diabetes. Estimates for the year 2000 and projections for 2030. Diabetes Care 27: 1047–1053. 15111519 10.2337/diacare.27.5.1047

[2] DeFronzo RA, Abdul-Ghani M (2011) Type 2 Diabetes can be prevented with early pharmacological intervention. Diabetes Care 34: S202–S209. 21525456 10.2337/dc11-s221 PMC3632162

[3] Ryder REJ (2011) Pioglitazone: an agent which reduces stroke, myocardial infarction and death and is also a key component of the modern paradigm for the optimum management of type 2 diabetes. The British Journal of Diabetes & Vascular Disease 11: 113–120.

[4] Colca JR, Kletzien RF (2006) What has prevented the expansion of insulin sensitisers? Expert Opin. Investig. Drugs 15: 205–210. 10.1517/13543784.15.3.205 16503758

[5] Kahn BB, McGraw TE (2010) Rosiglitazone, PPARγ, and type 2 diabetes. N Engl J Med 363: 2667–2669. 21190462 10.1056/NEJMcibr1012075 PMC3733169

[6] Chen Z, Vigueira PA, Chambers KT, Hall AM, Mitra MS, et al (2012) Insulin resistance and metabolic derangements in obese mice are ameliorated by a novel peroxisome proliferator-activated receptor γ –sparing thiazolidinedione. J Biol Chem 287: 23537–23548. 22621923 10.1074/jbc.M112.363960 PMC3390629

[7] Colca JR, VanderLugt JT, Adams WJ, Shashlo A, McDonald WG, et al (2013) Clinical proof of concept with MSDC-0160, a prototype mTOT modulating insulin sensitizer,. Clinical Pharmacology and Therapeutics 93: 352–359. 23462886 10.1038/clpt.2013.10 PMC3604641

[8] Feinstein DL, Spagnolo A, Akar C, Weinberg G, Murphy P, et al (2005) Receptor-independent actions of PPAR thiazolidinedione agonists: is mitochondrial function the key? Biochem. Pharmacol 70: 77–88. 10.1016/j.bcp.2005.03.033 15925327

[9] Colca JR, McDonald WG, Waldon DJ, Leone JW, Lull JM, et al (2004) Identification of a novel mitochondrial protein (“mitoNEET”) cross-linked specifically by a thiazolidinedione photoprobe. Am J Physiol Endocrinol Metab 286: E252–E260. 14570702 10.1152/ajpendo.00424.2003

[10] Colca JR, Harrigan GG (2004) Photo-Affinity labeling strategies in identifying the protein ligands of small molecules: Examples of targeted synthesis of drug analog photoprobes. Combinatorial Chemistry and High Throughput Screening 7: 699–704. 15578932 10.2174/1386207043328337

[11] Bricker DK, Taylor EB, Schell JC, Orsak T, Boutron A, et al (2012) A mitochondrial pyruvate carrier required for pyruvate uptake in yeast, *Drosophila*, and humans. Science 337: 96–100. 22628558 10.1126/science.1218099 PMC3690818

[12] Herzig S, Raemy E, Montessuit S, Veuthey JL, Zamboni N, et al (2012) Identification and functional expression of the mitochondrial pyruvate carrier. Science 337: 93–96. 22628554 10.1126/science.1218530

[13] Benga G, Hodarnau A, Tilinca R, Porutiu D, Dancea S, et al (1979) Fractionation of Human Liver Mitochondria: Enzymic and Morphological Characterization of the Inner and Outer Membranes as Compared to Rat Liver Mitochondria. J Cell Sci 35: 417–429. 422680 10.1242/jcs.35.1.417

[14] Wiley SE, Murphy AN, Ross SA, van der Geer P, Dixon JE (2007) MitoNEET is an iron-containing outer mitochondrial membrane protein that regulates oxidative capacity. Proc Natl Acad Sci USA 104: 5318–5323. 17376863 10.1073/pnas.0701078104 PMC1838440

[15] Musselman LP, Fink JL, Narzinski K, Ramachandran PV, Hathiramani SS, et al (2011) A high-sugar diet produces obesity and insulin resistance in wild-type Drosophila. Dis Model Mech 4 6: 842–849. 21719444 10.1242/dmm.007948 PMC3209653

[16] Petrovic N, Shabalina IG, Timmons JA, Cannon B, Nedergaard J (2008) Thermogenically competent nonadrenergic recruitment in brown preadipocytes by a PPARγ agonist. Am J Physiol Endocrinol Metab 295: E287–E296. 18492776 10.1152/ajpendo.00035.2008

[17] Loren MA, Burant CF, Kennedy RT (2011) Reducing time and increasing sensitivity in sample preparation for adherent mammalian cell metabolomics. Anal Chem 8: 3406–14. 10.1021/ac103313x PMC3094105 21456517

[18] Foellmi-Adams LA, Wyse BM, Herron D, Nedergaard J, Kletzien RF (1996) Induction of uncoupling protein in brown adipose tissue. Synergy between norepinephrine and pioglitazone, an insulin-sensitizing agent. Biochem Pharmacol 52: 693–701. 8765467 10.1016/0006-2952(96)00345-0

[19] Choi JH, Banks AS, Estall JL, Kajimura S, Bostrom P, et al (2010) Anti-diabetic drugs inhibit obesity-linked phosphorylation of PPARgamma by Cdk5. Nature 466 7305: 451–456. 20651683 10.1038/nature09291 PMC2987584

[20] Newgard CB (2012) Interplay between Lipids and Branched-Chain Amino Acids in Development of insulin resistance. Cell Metabolism 14: 606–614. 10.1016/j.cmet.2012.01.024 PMC3695706 22560213

[21] Brun RP, Spiegelman BM (1997) PPAR gamma and the molecular control of adipogenesis. J Endocrinol 155: 217–218. 9415052 10.1677/joe.0.1550217

[22] Sauermann M, Hahne F, Schmidt C, Majety M, Rosenfelder H, et al (2007) High-throughput flow cytometry–based assay to identify apoptosis-inducing proteins. J Biomol Screen 12: 510–520. 17478479 10.1177/1087057107301271

[23] Wiley SE, Paddock ML, Abresch EC, Gross L, van der Geer P, et al (2007) The outer mitochondrial membrane protein mitoNEET contains a novel redox-active 2Fe-2S cluster. J Biol Chem 282: 23745–23749. 17584744 10.1074/jbc.C700107200

[24] Paddock MA, Wiley S, Axelrod HL, Cohen AE, Roy M, et al (2007) MitoNEET is a uniquely folded 2Fe–2S outer mitochondrial membrane protein stabilized by pioglitazone. Proc Natl Acad Sci USA 104: 14342–14347. 17766440 10.1073/pnas.0707189104 PMC1963346

[25] Zuris JA, Ali SS, Yeh H, Nguyen TA, Nechushtai R, et al (2012) NADPH inhibits [2Fe-2S] cluster protein transfer from diabetes drug target MitoNEET to an Apo-acceptor protein. J Biol Chem 287: 11649–11655. 22351774 10.1074/jbc.M111.319731 PMC3320914

[26] Kusminski CM, Holland WL, Sun K, Park J, Spurgin SB, et al (2012) MitoNEET-driven alterations in adipocyte mitochondrial activity reveal a crucial adaptive process that preserves insulin sensitivity in obesity. Nat Med September 9. 10.1038/nm.2899 PMC3745511 22961109

[27] Satapati S, Sunny NE, Kucejova B, Fu X, He TT, et al (2012) Elevated TCA cycle function in the pathology of diet-induced hepatic insulin resistance and fatty liver. J Lipid Res 53: 1080–1092. 22493093 10.1194/jlr.M023382 PMC3351815

[28] Sunny NE, Parks EJ, Browning JD, Burgess SC (2011) Excessive hepatic mitochondrial TCA cycle and gluconeogenesis in humans with nonalcoholic fatty liver disease. Cell Metab 14: 804–810. 22152305 10.1016/j.cmet.2011.11.004 PMC3658280

[29] Halestrap AP (2012) The mitochondrial pyruvate carrier: has it been unearthed at last? Cell Metab 16: 141–143. 22883228 10.1016/j.cmet.2012.07.013

[30] Divakaruni AS, Wiley SE, Rogers GW, Andreyev AY, Petrosyan, et al (2013) Thiazolidinediones are acute, specific inhibitors of the mitochondrial pyruvate carrier. Proc Natl Acad Sci USA 110 14: 5422–5427. 23513224 10.1073/pnas.1303360110 PMC3619368

